# Poleward Range Shifts of Breeding Birds in Wisconsin

**DOI:** 10.1002/ece3.71796

**Published:** 2025-07-28

**Authors:** Drake T. Stallworth, Nicholas M. Anich, Benjamin Zuckerberg

**Affiliations:** ^1^ University of Wisconsin‐Madison Madison Wisconsin USA; ^2^ Wisconsin Department of Natural Resources Ashland Wisconsin USA

**Keywords:** breeding bird atlas, climate change, range boundaries, range contraction, range expansion, range shifts

## Abstract

Climate change is causing shifts in the geographic ranges of multiple species across the world. Birds are a critical taxon for observing range shifts because their high mobility allows them to track shifting climates over time and space. Breeding bird atlases are large‐scale breeding bird surveys that are essential for tracking species' ranges in response to climate change. In this paper, our goal was to analyze Wisconsin's Breeding Bird Atlases (1995–2000 and 2015–2019) to document changes in range size, mean latitude, and range boundary shift of breeding birds. Concordant with our predictions, we found that many southerly bird species with northern range limits were both expanding in distribution and shifting their range boundaries poleward. Contrary to our predictions, northerly species with southern range limits in the state were not shifting their boundary poleward but demonstrated a northward movement in their mean latitudes. Despite the lack of range boundary shift for northerly species as a group, many boreal species demonstrated a shift northward in their overall distribution. The repeated signal of bird ranges moving poleward remains a coherent fingerprint of the impact of modern climate change on species and ecosystems.

## Graphical abstract

Anthropogenic climate change has been documented to cause poleward range shifts in birds. This paper uses Wisconsin's two Breeding Bird Atlases to determine climate change's impact on the ranges of birds in the state. We found that warm‐adapted birds in the state were expanding northward.

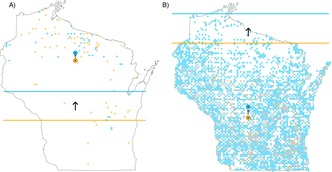

## 1 Introduction

The geographic range of a species can be conceptualized as the region in which a species occurs in space and time (Brown et al. 1996). What determines the limits of this range is a combination of factors but ultimately relies on a species' ecological niche and the environmental conditions that help a species fulfill it (Slatyer et al. 2013). These can include biotic factors such as resource availability, dispersal limitations, and biotic interactions that mediate a species' likelihood of occurrence in geographic space (Freeman and Montgomery 2016; Järvinen and Väisänen 1979; Sexton et al. 2009). Likewise, abiotic factors, such as climate, can play a major role in constraining range boundaries (Cahill et al. 2014).

Thermoregulatory constraints and environmental requirements often limit the distribution of organisms to specific climate conditions (Auer and Martin 2013; Cadieux et al. 2020; McCauley et al. 2017; Oswald and Arnold 2012; Sechley et al. 2015). For some species, temperature and precipitation alone can act as primary factors on species range boundaries, at times outweighing biotic factors such as competition and vegetation (Cahill et al. 2014; Sexton et al. 2009; Virkkala 2016). Since climate is such an important determinant of species ranges, one of the most prominent signals of climate change is the observed impact it has on the shifts in the geographic ranges of species and communities (Parmesan and Yohe 2003).

A clade of organisms that is commonly used to observe this relationship is birds due to their relatively high detectability and capacity to track shifting climate conditions (Devictor et al. 2008, 2012; Lenoir and Svenning 2015; Parmesan and Yohe 2003). Bird ranges have been used to observe climate change impacts through range boundary shift analysis (Brommer et al. 2012; Thomas and Lennon 1999; Zuckerberg, Woods, and Porter 2009). Many of these prior studies have found that both cold‐ and warm‐adapted species will shift poleward over time to track shifting climatic gradients associated with rising global temperatures. However, this pattern is not universal, as others have found some species shifting in other directions (Zurell et al. 2024) or shifts driven by factors other than climate (Venne and Currie 2021). Regardless of causality, range boundaries are the primary currency for exploring range shifts because they are most sensitive to environmental changes and serve as a more sensitive indicator of species' responses to climate change (Chen et al. 2011; La Sorte and Thompson III 2007; Sexton et al. 2009).

Range boundary shift analyses are not without challenges. Methodological replication is difficult across different regions (Brommer et al. 2012). Many analyses of range boundary shifts are conducted on the analysis of historical surveys collected at local scales and are challenging to fully capture range boundaries. To account for these shortcomings, community science initiatives, such as Breeding Bird Atlases, are of critical importance for quantifying range shifts. Most atlases are standardized in their data collection, so there is potential for replication among a diversity of geographic regions. Typically, atlases divide a state, province, or country into a grid of blocks with species occupancy and breeding status being recorded in each (Jackson et al. 1996). The result is a standardized method of tracking multi‐species data at regional scales. Additionally, atlases can be repeated over time, offering a powerful tool for delineating range changes across the designated region. Such characteristics of atlases make them ideal for conducting range boundary shift analyses.

Wisconsin is a state that is well‐suited for exploring geographic changes in ranges for multiple bird species. When assessing range boundary shifts, it is commonplace to classify bird species into northerly and southerly categories based on the limit of the species' range within the state (Brommer et al. 2012; Thomas and Lennon 1999; Zuckerberg, Woods, and Porter 2009). Wisconsin is unique in that it is characterized by an ecological transition area that separates the northern coniferous forests from the southern hardwood forests and prairies called the Tension Zone (Curtis 1959). Many Wisconsin breeding bird species have range boundaries that coincide with the Tension Zone (Robbins 1991), that makes Wisconsin a prime candidate to analyze the shifts in these naturally occurring limits. Additionally, northern Wisconsin harbors several boreal breeding birds, species that are exceptionally susceptible to a warming climate (Jiguet et al. 2010; Virkkala and Rajasärkkä 2011). The presence of a boreal ecosystem makes it especially important to observe how climate affects Wisconsin's bird communities.

In this paper, our goal was to analyze Wisconsin's two Breeding Bird Atlases to quantify the overall shift in range boundaries for northerly and southerly bird species in the state over a 20‐year period. Along with range boundaries, we analyzed change in range size and shift in mean latitude to provide a clearer understanding of species' range changes. We separately analyzed a subset of the northerly boreal bird species due to the sensitivity of the boreal ecosystem to a warming climate. Under the broader hypothesis of poleward range shifts, we predicted that northerly, southerly, and boreal birds would shift their boundaries northward over the last 20 years as well as their mean latitudes, but to a lesser degree due to the sensitivity of range boundaries to environmental change. Based on other atlas studies, we further predicted that southerly bird species would display the most dramatic shift (Brommer et al. 2012; Thomas and Lennon 1999) because colonization of a new block requires just one new record in that area, whereas extirpation (expected along the southern edge of the northerly species) requires zero detections in that block for the entire multi‐year atlasing period. Through this study, we seek to understand climatic responses of Wisconsin's breeding bird community to better dictate management to remedy its effects.

## 2 Methods

### 2.1 The Wisconsin Breeding Bird Atlas

The Wisconsin Breeding Bird Atlas (WBBA) is a comprehensive, statewide survey designed to document occupancy and abundance of all the bird species nesting in the state. WBBA surveys occurred during two separate time periods: 1995–2000 and 2015–2019. The atlas followed standardized protocols where volunteers visited a block multiple times during the breeding season with the goal of documenting breeding for all species within the block (Beck et al. 2018). Atlas volunteers attended statewide multi‐day training sessions, although training material was also available online and all observations from any observer were accepted following multiple phases of expert review.

Each bird observed was recorded along with breeding codes describing four different levels of breeding evidence. The categories were: observed (no evidence for breeding), possible (singing birds and birds present in suitable habitat), probable (e.g., territorial defense), and confirmed (e.g., nests with young) (Cutright et al. 2006).

Atlas blocks were one‐sixth of a U.S. Geological Survey 7.5‐min topographic quadrangle, approximately 4.8 km × 4.8 km in size (23 km^2^; USGS 2020; Wisconsin DNR 2024). One block in each quadrangle (generally the center east block) was designated a priority block that required coverage, which ensured standardized statewide coverage during both atlas periods (Cutright et al. 2006). There were 6644 total blocks in the full sampling grid across Wisconsin. During the first atlas 1156 were deemed priority blocks that required coverage during the first atlas (Cutright et al. 2006). The second atlas retained all first atlas priority blocks and added additional priority blocks to make 1283 priority blocks that required coverage during the second atlas. See Supporting Information: Appendices A and B for visualizations of full coverage and priority blocks during each atlas period.

There were no specific restrictions on atlasing within a block, but atlasers were instructed to address the following six criteria to ensure complete coverage in atlas priority blocks: (1) at least 20 h of effort spent in a block over multiple surveys; (2) at least 80% of species found during Atlas I were detected (for the second atlas; the first atlas used an estimate of projected species); (3) at least 50% of species detected (i.e., coded as at least possible) were confirmed as breeding; (4) all habitat types within the block have been visited; (5) surveys were completed at different times of year; and (6) at least two nocturnal visits occurred. The atlas accepted records year‐round, though there were relatively few breeding species nesting September–March and most records were submitted from April through August, with June and July the most active months. For both atlases, submissions were screened using existing eBird filters that flagged birds unusual for their date and location. Data for both atlases were also screened by an expert panel for accuracy based on range, time of year, and breeding code for each species.

For the purposes of this study, we chose 84 species that regularly breed in Wisconsin. Species had to be observed in at least 10 atlas blocks in both atlases and had to have a range boundary in Wisconsin. We classified the 84 species into a “Northerly” or “Southerly” category based on their mapped atlas distribution (eBird 2025). We classified bird species with breeding ranges with a southern range limit within Wisconsin as “Northerly” and species with breeding ranges with a northern range limit within Wisconsin as “Southerly”. In most cases these classifications correspond to species whose continental breeding range is north or south of Wisconsin respectively, but for certain species their continental range is broader, for example a few Southerly species also range into the northern Great Plains, and a few Northerly species extend south into Appalachia. For our analysis of boreal species, we classified a subset of Northerly species as being a “Boreal species” if they bred in Wisconsin's boreal coniferous habitat (Fink et al. 2023).

For this analysis, we obtained atlas data from eBird (2023) and then filtered the data in several ways. We limited the dataset to only records with breeding codes (i.e., possible, probable or confirmed codes) to exclude out‐of‐season observations or any records deemed to not be breeding locally. We removed any duplicate occurrences of a species in each block to limit the data to occupancy. Because Atlas I data was collected at the block level, we shifted all locations from Atlas II to block mean latitudes as well.

### 2.2 Range Size Change Analysis

We quantified three metrics: (1) change in range size, (2) shift in range boundary, and (3) shift in mean latitude (Brommer et al. 2012; Thomas and Lennon 1999). Change in range size reflected range expansion or contraction from Atlas I to Atlas II. Sampling intensity and coverage were slightly higher in the second atlas (although there was no geographic shift in coverage) (Supporting Information: Appendix C). To correct for slight changes in coverage between the atlases, we limited the range size analysis to a set of comparable blocks that were covered well during both atlas periods. To create the set of comparable blocks, we first limited the dataset to blocks which were Atlas II priority blocks, which we knew were covered well. We then used breeding species richness (i.e., the number of species coded as at least possible in a block) as a proxy to weed out blocks with insufficient survey effort (Kujala et al. 2013). Specifically, we calculated the ratio of the number of breeding‐coded species in the second Atlas to the number of breeding‐coded species in the first Atlas, and blocks with a ratio lower than 0.75 or higher than 1.25 were removed (Herrando et al. 2020). This set of 858 comparable blocks (Supporting Information: Appendix D) was then used to measure the overall difference in the number of occupied blocks for each species between the two atlases: (i.e., No. of blocks in Atlas II – No. of blocks in Atlas I). The resulting difference was then divided by the number of occupied blocks in the first atlas, then converted to a percentage: [(No. of blocks in Atlas II – No. of blocks in Atlas I)/(No. of blocks in Atlas I) × 100]. We used a Pearson's chi‐squared test using the R statistical platform (R Core Team 2024) to determine the significance of each individual species' change in range size as well as the proportion of species in each assemblage that was contracting or expanding (Greenwood and Nikulin 1996).

### 2.3 Mean Latitude and Range Boundary Shift Analysis

Shifts in mean latitude reflect the direction and magnitude in which the average latitude of a species shifted, whereas shifts in range boundaries reflect the direction and magnitude in which the northern or southern boundary of a species shifted between the two Atlases. For the mean latitude and boundary analyses, we used all detections from both atlases because many times the outermost boundary records were not in our restricted set of comparable blocks, and limiting this analysis to only 858 of 6644 blocks would unduly remove relevant information.

To measure overall mean latitude shifts, we calculated the mean latitude for all species in both atlases and conducted a non‐paired Mann–Whitney *U* test to determine the significance in the geographic shift of each individual species (Dixon and Massey 1951; R Core Team 2024). We also conducted a paired Wilcoxon signed rank sum test (Dixon and Massey 1951) to determine the significance of the mean shift for the entire assemblage (Northerly, Southerly, and Boreal). We also recorded the species‐specific differences in mean latitudes between atlases.

To measure range boundary shifts, we calculated the mean latitude of the most southerly 10 atlas blocks for Northerly species to represent their trailing edge and the mean latitude of the top 10 most northerly blocks for Southerly species to represent their leading edge, as has been used in other studies (Thomas and Lennon 1999; Zuckerberg, Woods, and Porter 2009). We also re‐ran the analysis using 20, 30, and 40 blocks for delineating range boundaries (Supporting Information: Appendix E). We conducted a non‐paired Mann–Whitney *U* Test (Dixon and Massey 1951; R Core Team 2024) to determine the significance of the observed boundary shift for individual species and a paired Wilcoxon signed rank sum test (Dixon and Massey 1951; R Core Team 2024) to determine the significance of the observed boundary shifts for each assemblage. A Pearson's chi‐squared test for count data was conducted using R Studio (R Core Team 2024) to determine the proportion of each assemblage that was shifting poleward or southward (Greenwood and Nikulin 1996). Further, we implemented a general linear model (GLM) assuming a non‐normal distribution (Penny et al. 2007; Thomas and Lennon 1999) where change in range size was included as the predictor and range boundary shift (km) as the response variable. As was done with previous studies, we used the change in number of occupied blocks for each atlas (log_10_ transformed) (Brommer et al. 2012; Thomas and Lennon 1999; Zuckerberg, Woods, and Porter 2009). Using the GLM, we estimated the *y*‐intercept to determine the shift in range boundary while controlling for overall changes in range size (Thomas and Lennon 1999). Boreal species had relatively few detections, which prevented us from doing regression analysis of boundary shifts, so we focused only on the mean latitude approach. All analyses were performed using the R statistical platform (R Core Team 2024).

## 3 Results

### 3.1 Change in Range Size

Of 84 bird species included in our analysis, 53 species were classified as Northerly while 31 were classified as Southerly (Supporting Information: Appendices S1 and G). When considering all Northerly species, the ratio of species that were expanding and contracting was not significantly different (*χ* ^2^ = 0.019, *p* = 0.89) with an average range expansion of 9.0%. For species that showed a statistically significant (*p* < 0.05) change in range size, 18.9% of Northerly species were expanding while 26.4% were contracting (Table 1; Supporting Information: Appendix H). Trumpeter Swan ( *Cygnus buccinator* ) had the largest range expansion with a 573.3% increase (*p* < 0.05) between both atlases (Table 1). Other species with notable and significant (*p* < 0.05) range expansions were Merlin ( *Falco columbarius* ) (339.5%), Bald Eagle ( *Haliaeetus leucocephalus* ) (112.0%), and Pine Warbler ( *Setophaga pinus* ) (51.2%) (Table 1). Out of the 53 Northerly species, 20 species were classified as boreal species (Fink et al. 2023), where 45% were contracting, including large and significant (*p* < 0.05) range contractions in Evening Grosbeak ( *Coccothraustes vespertinus* ) (91.8% decrease), Boreal Chickadee ( *Poecile hudsonicus* ) (81.8%), Connecticut Warbler ( *Oporornis agilis* ) (81.6%), and Canada Jay ( *Perisoreus canadensis* ) (80.3%) (Table 1; Supporting Information: Appendix H).

**TABLE 1:** The percent change in range, boundary shift (km), and shift in mean latitude (km) of Northerly species between the first (1995–2000) and second (2015–2019) Wisconsin breeding bird atlases.

Species	% Range increase	Boundary shift (km)	Mean latitude shift (km)
Trumpeter Swan	573.33*	−127.29*	−14.85
American Black Duck	−75.47*	100.45*	22.65
Common Merganser	−20.63	−13.89	21.65*
Ruffed Grouse	−20.89*	20.83*	37.32*
Common Loon	4.40	−0.93	−0.60
Osprey	45.86*	−100.45*	−53.18*
Sharp‐shinned Hawk	−50.00*	65.27*	23.46*
**American Goshawk**	−20.00	92.12*	39.87*
Bald Eagle	111.96*	−30.59*	−59.86*
Broad‐winged Hawk	7.41	−25.46*	−3.47
Yellow‐bellied Sapsucker	29.42*	−15.91*	−11.30*
**Black‐backed Woodpecker**	−75.86*	49.53*	17.40
Pileated Woodpecker	35.53*	−10.84*	3.64
Merlin	339.47*	−235.16*	−55.59*
**Olive‐sided Flycatcher**	−66.67*	16.20	2.79
**Yellow‐bellied Flycatcher**	−1.90	−27.77*	−0.60
**Alder Flycatcher**	17.27*	−8.80	9.75
Least Flycatcher	−5.08	6.48*	23.8*
Blue‐headed Vireo	18.56	−23.61	4.53
**Canada Jay**	−80.26*	30.55*	6.78
Common Raven	26.51*	−48.61*	−14.56*
**Boreal Chickadee**	−81.82*	16.66*	14.06
**Ruby‐crowned Kinglet**	−36.07*	−16.56*	5.40
Golden‐crowned Kinglet	11.39	−31.94	4.48
Brown Creeper	10.65	−5.45	19.45*
**Winter Wren**	−16.97*	−4.17	18.62*
Veery	5.09	−15.51*	16.82*
**Swainson's Thrush**	−62.79*	69.90	48.84*
Hermit Thrush	4.93	−18.05*	0.85
**Evening Grosbeak**	−91.79*	56.01*	8.79
Purple Finch	8.13	10.18	6.28
Dark‐eyed Junco	−39.62*	31.48*	10.27
**White‐throated Sparrow**	−3.93	2.78	12.93*
LeConte's Sparrow	−28.57	14.81	58.35*
**Lincoln's Sparrow**	−1.98	−54.62*	2.11
Ovenbird	−1.81	−0.93	15.46*
**Northern Waterthrush**	24.00*	24.53*	27.80*
Golden‐winged Warbler	−12.10	52.31*	20.38*
Black‐and‐white Warbler	3.46	11.11	11.81*
Nashville Warbler	−0.24	6.02	13.39*
**Connecticut Warbler**	−81.58*	71.29*	8.74
Mourning Warbler	−12.22*	−4.17	25.42*
**Cape May Warbler**	21.43	−30.09*	−2.37
Northern Parula	3.47	−153.01*	0.99
**Magnolia Warbler**	−2.11	69.90*	17.47*
**Blackburnian Warbler**	16.99	55.09*	9.51
Chestnut‐sided Warbler	1.73	−6.02	15.10*
Black‐throated Blue Warbler	6.45	5.56	9.69
**Palm Warbler**	2.17	−52.77*	−7.47
Pine Warbler	51.16*	−39.81*	−3.77
Yellow‐rumped Warbler	10.03	10.65	7.17
Black‐throated Green Warbler	−5.10	22.68	18.21*
**Canada Warbler**	−17.56	42.13*	34.86*

*Note:* Positive shift values indicate northward shifts. Boreal birds are indicated in bold, and significant (*p* < 0.05) shifts are designated with “*”. Confidence intervals are reported in Supporting Information: Appendix I.

For all Southerly species, we found that 71.0% of species were expanding while 29.0% of their ranges were contracting (*χ* ^2^ = 5.45, *p* < 0.05) with an average range expansion of 47.2%. Considering only the species that showed a statistically significant change in range size, 48.4% were expanding and 3.2% were contracting (Supporting Information: Appendix H). Lark Sparrows ( *Chondestes grammacus* ) had the largest range expansion with a 246.7% increase (*p* < 0.05) from the previous atlas (Table 2). Other species that demonstrated significant (*p* < 0.05) range expansions were Orchard Oriole ( *Icterus spurius* ) (235.4%), Tufted Titmouse ( *Baeolophus bicolor* ) (188.2%), and Dickcissel ( *Spiza americana* ) (162.1%) (Table 2). Meanwhile, Purple Martin ( *Progne subis* ) was the only Southerly species that was contracting its range with a 35.0% decrease (*p* < 0.05) (Table 2).

**TABLE 2:** The percent expansion in range, boundary shift (km), and mean latitude (km) of Southerly species between the first (1995–2000) and second (2015–2019) Wisconsin breeding bird atlases.

Species	% Range change	Boundary shift (km)	Mean latitude shift (km)
Wild Turkey	97.76*	110.64*	67.77*
Ring‐necked Pheasant	4.56	43.82*	46.24*
Yellow‐billed Cuckoo	35.29*	17.00	0.84
Sandhill Crane	47.79*	24.53*	19.34*
Red‐shouldered Hawk	18.42	0.93	4.32
Eastern Screech‐Owl	9.91	48.14	6.69
Red‐headed Woodpecker	−13.22	33.79*	−1.98
Red‐bellied Woodpecker	43.31*	87.66*	33.00*
Acadian Flycatcher	41.38*	90.52*	3.72
Willow Flycatcher	10.53	−10.18	−2.53
Bell's Vireo	44.44	7.87	1.76
Yellow‐throated Vireo	25.05*	1.39	6.00
Tufted Titmouse	188.24*	52.77*	23.01*
Horned Lark	−6.63	6.02	1.43
Purple Martin	−35.00*	−51.38*	−9.41
Blue‐gray Gnatcatcher	17.90*	23.26*	−1.86
Carolina Wren	133.33*	110.17*	−17.61
Wood Thrush	7.62	13.42*	23.58*
House Finch	−11.43	4.99	−17.96*
Grasshopper Sparrow	−17.99	3.24	9.68
Field Sparrow	−1.24	25.00*	−7.71
Lark Sparrow	246.67*	123.14*	1.29
Henslow's Sparrow	89.09*	81.47*	−14.68
Orchard Oriole	235.35*	57.40*	24.25*
Louisiana Waterthrush	−11.54	23.12	−16.89
Blue‐winged Warbler	23.21*	32.35*	30.06*
Prothonotary Warbler	−3.45	44.42*	−31.61*
Hooded Warbler	85.71*	12.44	−10.50
Cerulean Warbler	−10.17	−4.23	−22.44*
Northern Cardinal	6.09	63.42*	11.42*
Dickcissel	162.09*	101.38*	40.48*

*Note:* Positive shift values indicate northward shifts. Significant shifts and increases are designated with “*”, and confidence intervals are reported in Supporting Information: Appendix J.

### 3.2 Mean Latitude Shift Analysis

Overall, 77.4% of Northerly species were shifting their mean latitudes northward while 22.6% were moving southward (*χ* ^2^ = 15.87, *p* < 0.05) with an average shift in mean latitude of 8.5 km (*V* = 281, *p* < 0.05). Considering only the species that showed a statistically significant shift, 39.6% were shifting northward and 9.4% were shifting southward. When analyzing the shifts in mean latitude, the Northerly species with the most dramatic poleward shift was LeConte's Sparrow ( *Ammospiza leconteii* ) (58.4 km, *p* < 0.05) (Table 1). Other Northerly species with significant (*p* < 0.05) poleward shifts were Swainson's Thrush ( *Catharus ustulatus* ) (48.8 km), American Goshawk (39.9 km), and Ruffed Grouse ( *Bonasa umbellus* ) (37.3 km) (Table 1). The Northerly species with significant southward mean latitude shifts included Bald Eagle (59.9 km), followed by Merlin (55.6 km), Osprey (53.2 km), and Common Raven ( *Corvus corax* ) (14.6 km) (Table 1), all of which had expanding ranges.

**FIGURE 1:**
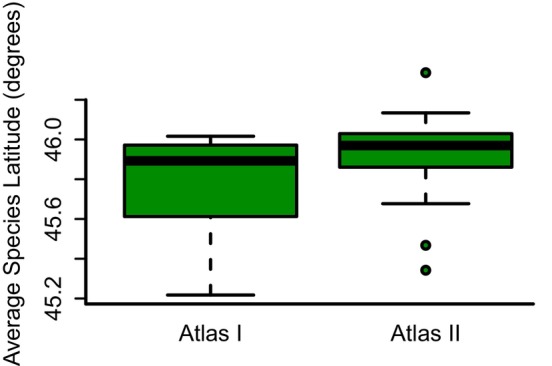
Average latitudes of the subset of Northerly species that breed in the boreal region of Wisconsin for both Atlas I and Atlas II. Boreal species had an overall significant poleward shift in mean latitude of 13.8 km.

Among boreal species, the most northward shift in mean latitude was Swainson's Thrush (48.8 km, *p* < 0.05) followed by American Goshawk (39.9 km, *p* < 0.05), Canada Warbler ( *Cardellina canadensis* ) (34.9 km, *p* < 0.05), and Northern Waterthrush (*Parkesia novaboracensis*) (27.8 km, *p* < 0.05) (Table 1). Considering all boreal species, 85.0% of boreal birds were shifting their mean latitudes northward while 15.0% were moving southward (*χ* ^2^ = 9.80, *p* < 0.05). Considering only the species that showed a statistically significant shift, 35.0% were moving northward while no boreal bird species were moving southward. The average shift in mean latitude of the boreal species was 13.6 km poleward (*V* = 11, *p* < 0.05), suggesting an overall poleward movement (Figure 1).

**FIGURE 2:**
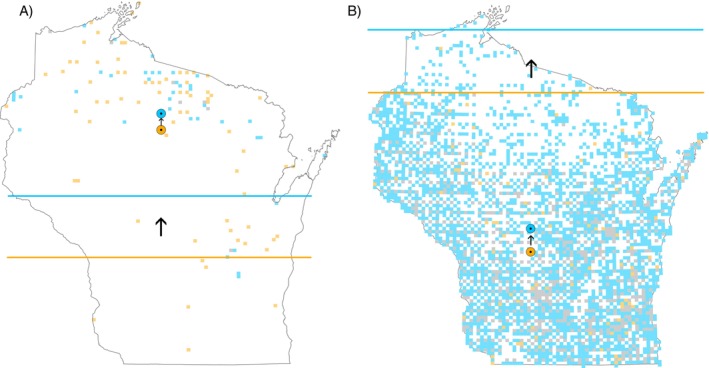
Example change in range size, boundary shift, and shift in mean latitude for (A) American Black Duck ( *Anas rubripes* ) and (B) Red‐bellied Woodpecker ( *Melanerpes carolinus* ). Orange blocks represent the blocks occupied in only Atlas I, blue blocks represent the blocks occupied in only Atlas II, and gray blocks were occupied during both atlas periods. We include all records from both atlases. The orange line represents the mean latitude of 10 boundary blocks in Atlas I, and the blue line represents this boundary in Atlas II. The orange central dot represents the mean latitude of all records in Atlas I, and the blue central dot represents the mean latitude in Atlas II. The arrows represent the direction of shift for the boundary and mean latitude.

Overall, there was no significant difference between Southerly species shifting their mean latitudes northward and southward (*χ* ^2^ = 1.58, *p* = 0.21) with a nonsignificant average shift (*V* = 181, *p* = 0.20). Considering only the species that showed a statistically significant shift, 32.3% were shifting northward while 9.7% were shifting southward (*χ* ^2^ = 10.9, *p* < 0.05). The Southerly species with the most dramatic northward shift in mean latitude was Wild Turkey (67.8 km, *p* < 0.05). Other species with notable shifts were Ring‐necked Pheasant ( *Phasianus colchicus* ) (46.2 km, *p* < 0.05), Dickcissel (40.5 km, *p* < 0.05), and Red‐bellied Woodpecker (*Melanerpes carolinus, p* < 0.05) (33.0 km) (Table 2; Figure 2B). The Southerly species with the greatest southward shift in mean latitude was Prothonotary Warbler ( *Protonotaria citrea* ) (31.6 km, *p* < 0.05) followed by Cerulean Warbler (22.4 km, *p* < 0.05) and House Finch ( *Haemorhous mexicanus* ) (18.0 km, *p* < 0.05) (Table 2).

### 3.3 Range Boundary Shift Analysis

**FIGURE 3:**
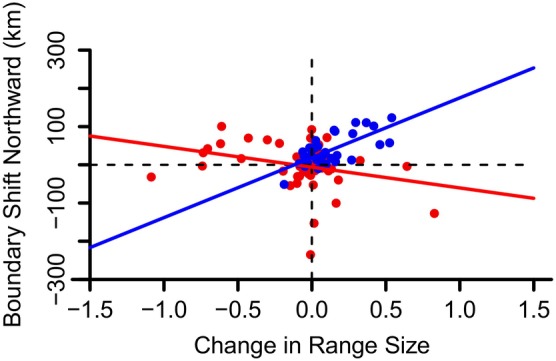
Change in range size (log_10_[No. of blocks in Atlas II] − log_10_[No. of blocks in Atlas I]) plotted against boundary shift (km) for both Northerly (red) and Southerly species (blue), with the *y*‐intercepts representing boundary shift with no expected range change size. Northerly species had an insignificant southward shift in their southern boundary whereas Southerly species had a significant and systematic poleward shift in their northern boundary.

Across all Northerly species, the proportion of species shifting their range boundaries northward and southward was not significantly different (*χ* ^2^ = 0.019, *p* = 0.89) with a nonsignificant boundary shift (*V* = 716, *p* = 0.82) (Supporting Information: Appendix H). Considering only the species that showed a statistically significant (*p* < 0.05) shift, 30.2% were shifting northward while 32.1% were shifting southward. Based on the GLM regression, the Northerly species had a non‐significant *y*‐intercept (*p* = 0.44) (Figure 3), suggesting no systematic shift in the latitude of their range boundaries after accounting for changes in range size. For individual species, American Black Duck ( *Anas rubripes* ) had the largest northward movement in its range boundary (100.5 km, *p* < 0.05) (Table 1; Figure 2A). Other species with notable and significant poleward range boundary shifts were American Goshawk (92.1 km) (*Astur atricapillus*), Connecticut Warbler (71.3 km), and Magnolia Warbler ( *Setophaga magnolia* ) (69.9 km). Merlin was the Northerly species that moved southward the most (235.2 km) (Table 1). Significant southward‐moving species were Northern Parula ( *Setophaga americana* ) (153.0 km), Trumpeter Swan (127.3 km), and Osprey ( *Pandion haliaetus* ) (100.5 km) (Table 1).

For all Southerly species, 90.3% were shifting their boundaries northward while 9.7% were moving southward (*χ* ^2^ = 20.16, *p* < 0.05) with an average boundary shift of 38.0 km north (*V* = 33, *p* < 0.05). Considering only species that showed a statistically significant shift, 58.1% were shifting poleward while 3.2% were shifting southward. When plotted against change in range size, Southerly species had a *y*‐intercept of 18.42 km (*p* < 0.05) (Figure 3). Lark Sparrow was the Southerly species that shifted the most in its range boundary (123.1 km, *p* < 0.05) (Table 2). Other notable and significant northward‐shifting species were Wild Turkey ( *Meleagris gallopavo* ) (110.6 km), Carolina Wren ( *Thryothorus ludovicianus* ) (110.2 km), and Dickcissel (101.4 km) (Table 2). Purple Martin was the only Southerly species that shifted its boundary significantly southward (51.4 km, *p* < 0.05) (Table 2).

## 4 Discussion

Our goal was to quantify range shifts for breeding birds in a region experiencing rapid environmental change over the past 20 years. By doing so, we aimed to further document evidence of poleward movements of breeding bird species. As a group, we found mixed evidence for a systematic retraction northward for northerly species. However, southerly species demonstrated a general range expansion that was congruent with our predictions, resulting in a systematic northward movement across many species range boundaries. Together, these results suggest that many birds in the Upper Midwest of the United States are shifting northward in their geographic ranges, but that most of this response is being driven by the expansion and colonization of new areas by southerly, warm‐adapted species.

For northerly birds, many species demonstrated a poleward shift in their mean latitude, but less of a response in their southerly range boundaries. The lack of a significant boundary shift could be because a range boundary shift for a northerly species is less likely to occur in comparison to a range boundary shift for a southerly species. For northerly species, range contraction relies on processes of extirpation occurring within an atlas block on their southern range edge and requires zero detections of the given species during the entire 5‐year atlasing period. Conversely, a single colonization record on the northerly edge for a southerly species contributes to a northward‐moving boundary. Birds are less willing to abandon breeding sites since being familiar with a site increases breeding success (Beletsky and Orians 1991; Bruinzeel and van de Pol 2004; Schlossberg 2009). Wisconsin's northerly birds may also be more varied in their habitat requirements, like the Merlin, which specializes on large nesting trees (Cava et al. 2014). Given that over 75% of northerly species shifted northward in their mean latitudes, however, suggests a potential shift that is being exhibited initially by shifts in abundance (Virkkala and Lehikoinen 2014).

As predicted, boreal species contracted northward between the two atlas periods. Many of these birds are more specialized in their habitat/climatic requirements (Cadieux et al. 2020; Wells and Blancher 2011), which would explain the greater magnitude of shift in mean latitude compared to the rest of the northerly bird community. The patterns observed with boreal species exemplify the conservation concern this assemblage represents. The sensitivity to climate paired with relatively few detections of boreal species led to the degree of range contraction being relatively higher than the rest of the northerly species. Additionally, considering the unstable nature of populations near a range limit (Jiguet et al. 2010; La Sorte and Thompson III 2007), several boreal species could be entirely extirpated from the state of Wisconsin by the third Atlas.

Southerly birds as a group demonstrated a significant northward shift in their range boundary. This was congruent with the findings of various other studies as colonization in the northern limit of southerly birds is far more likely than extirpation of the southern limit of northerly birds (Brommer et al. 2012; Hitch and Leberg 2007; Rodewald et al. 2016; Thomas and Lennon 1999). Such a response could also be due to the number of shared habitat requirements among the southerly group. Interestingly, the lack of shift in mean latitude suggests southerly species are expanding into the state while maintaining solid numbers in southern Wisconsin, emblematic of a community that is highly persistent in a warming climate (Lenoir and Svenning 2015).

Southerly species shifting northward may result in wholesale changes in community composition as warm‐adapted species increase in prevalence and abundance (Princé and Zuckerberg 2015). As species shift their distributions throughout the state, interspecific competition among previously isolated groups of birds may come into play (Freeman and Montgomery 2016; Järvinen and Väisänen 1979). Shifts in both climate conditions and vegetative composition could lead to increased niche overlap (Auer and Martin 2013; Felton et al. 2013), and based on previous research, such overlaps tend to favor warm‐adapted species, a process known as “thermophilization” (Gottfried et al. 2012), which would accelerate the poleward shift of the northerly bird community (Bocedi et al. 2013; Freeman and Montgomery 2016; Liu et al. 2024; Princé and Zuckerberg 2015).

The range contraction of northerly species due to a warming climate has been associated with their decline in regions with geographical barriers that limit where a species can go (Brommer et al. 2012; Laaksonen and Lehikoinen 2013; Virkkala 2016). Even with no barrier limiting movement, populations may be unable to keep pace with rising temperatures, and reproductive output can diminish as their environment becomes more unsuitable (Lauck et al. 2023; McCauley et al. 2017; Northrup et al. 2019). These declines can be exacerbated by interbreeding with the less productive populations on the edge of their range (Kirkpatrick and Barton 1997). Range shifts have implications for a species' population demographics, and many northerly bird species that demonstrated range shifts may be facing mounting conservation concerns. Further study should investigate causal factors of range shifts to better guide potential conservation action.

Despite boundary shift analysis being a widely used method, we acknowledge its limitations. As with many large‐scale monitoring programs, we detected a difference in effort and coverage between the two atlases. However, we found little evidence of geographic shifts in sampling coverage and focused on multiple metrics of range shifts. For overall changes in range size, we focused on only comparable priority blocks. We recognize that heterogeneous detection probabilities are likely across species, and methods (e.g., occupancy modeling) are available for accounting for detection probabilities, but we did not have information for when repeat visits occurred. Further, our results do not account for changes in within‐range densities, and it is possible that the more subtle changes in abundance may be undetected. However, previous studies have shown that changes in occurrence as measured by atlases are highly congruent with changes in abundance (Zuckerberg, Porter, and Corwin 2009). Birds have also been known to breed earlier to compensate for climate change without shifting their ranges (Socolar et al. 2017), and further research should focus on phenological responses of Wisconsin's breeding bird community. Finally, it is worth noting that additional directional analyses would be useful to relate observed range changes to more complex shifts in climate space and work to elucidate additional causal mechanisms.

Our results broadly corroborate the systematic shifts of species across other states and countries as it appears southerly species shifting poleward is a universal fingerprint of climate change. Wisconsin, like other midwestern regions, harbors a unique blend of warm‐ and cold‐adapted species meeting their distributional limits. It is within the regions where the expansion of warm‐adapted species may lead to a shift in community composition and novel biotic interactions. These novel interspecific interactions pave the way for shifting boundary limits and may force northerly species to shift poleward or decline in abundance. Further analysis including data from a third atlas could help describe these longer trends. Boreal species—an already restricted community in Wisconsin—are exhibiting a poleward shift and range contraction that could lead to many members of the assemblage being extirpated from the state and other regions in the future. We recommend that management efforts establish climate refugia along the southern range limit for boreal bird communities to provide suitable breeding conditions in a decreasingly suitable climate. We also recommend that monitoring efforts continue to anticipate climatic responses in birds and identify vulnerable species and communities.

## Author Contributions

**Drake T. Stallworth:** conceptualization (equal), data curation (equal), formal analysis (lead), investigation (lead), methodology (lead), writing – review and editing (lead). **Nicholas M. Anich:** conceptualization (equal), data curation (equal), resources (equal), writing – review and editing (equal). **Benjamin Zuckerberg:** funding acquisition (lead), methodology (lead), writing – review and editing (equal).

## Conflicts of Interest

The authors declare no conflicts of interest.

## Supporting information


Appendix S1.

## Data Availability

Data openly in Dryad at https://doi.org/10.5061/dryad.f1vhhmh70. Data for bird species was from Wisconsin Breeding Bird Atlas I and Wisconsin Breeding Bird Atlas II.
