# Supplementary material for: Poleward Range Shifts of Breeding Birds in Wisconsin
Source: Ecol Evol. 2025 Jul 28;15(8):e71796. doi: 10.1002/ece3.71796 (PMC12304433; doi:10.1002/ece3.71796)
Supplement: Supplementary file 1 — Appendix S1. [file ECE3-15-e71796-s001.docx]

**Appendix**


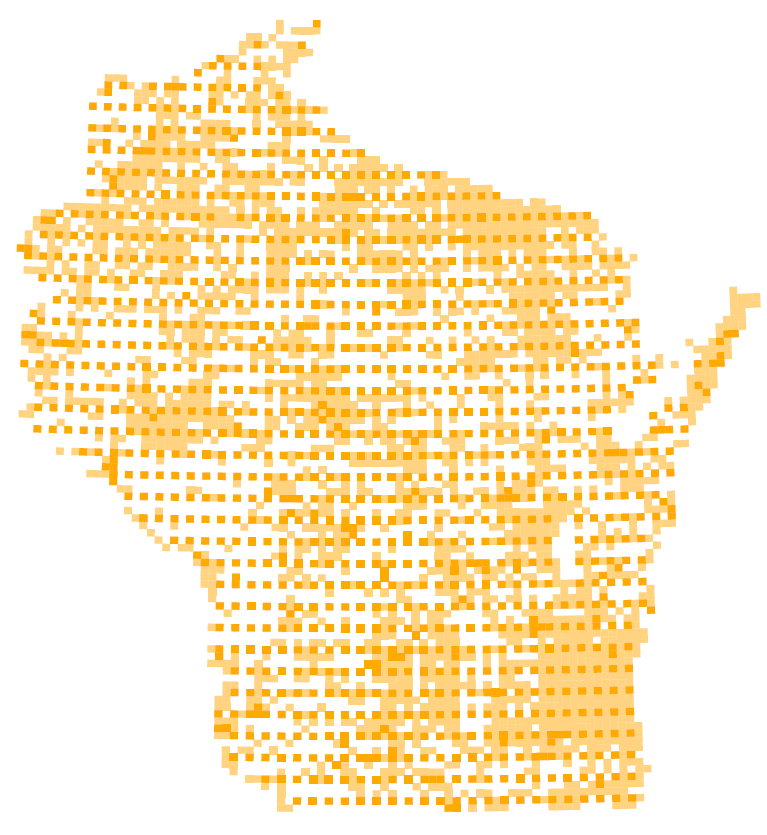


**Appendix A.** Block coverage during Wisconsin Breeding Bird Atlas I (1995–2000). Light orange blocks indicate at least one report from a block, and dark orange blocks indicate Priority blocks to which a high level of effort was directed (including 20 hours over multiple visits and 50% of breeding species confirmed as breeders).


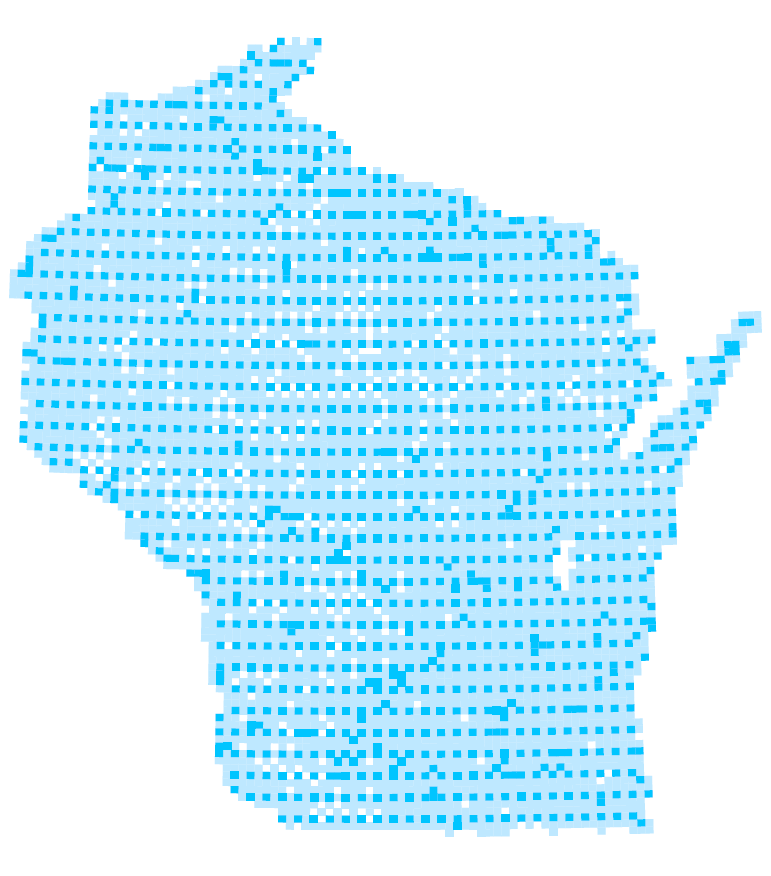


**Appendix B.** Block coverage during Wisconsin Breeding Bird Atlas II (2015–2019). Light blue blocks indicate at least one report from a block, and dark blue blocks indicate Priority blocks to which a high level of effort was directed (including 20 hours over multiple visits and 50% of breeding species confirmed as breeders).


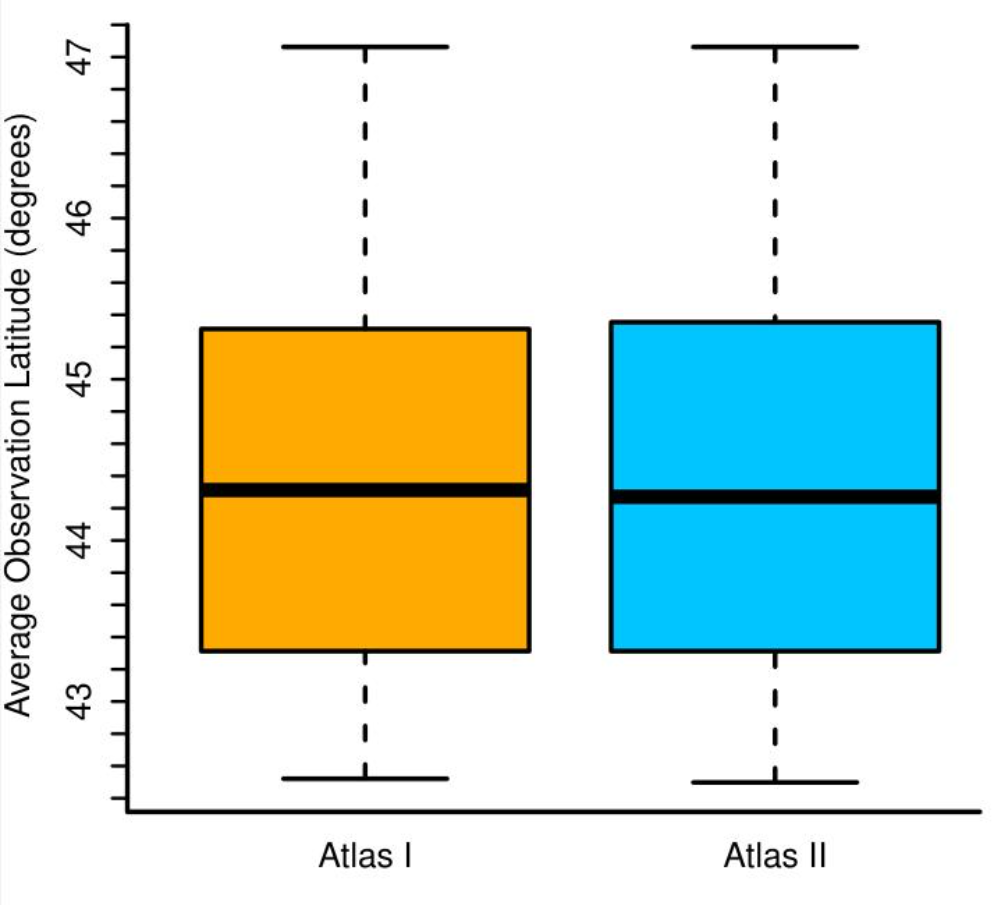


**Appendix C.** These boxplots depict the mean latitude of sightings for both periods of the Wisconsin Breeding Bird Atlas. Records were filtered to a maximum of one visit per block per day (First atlas n=32,192; Second atlas n=118,100), and second atlas latitudes were converted to block centroids to match the first atlas resolution. The mean latitude values were 44.39 N for the first atlas, and 44.37 N for the second atlas, indicating despite an overall difference in the amount of data between atlas periods, the latitudinal distribution of coverage was quite similar.


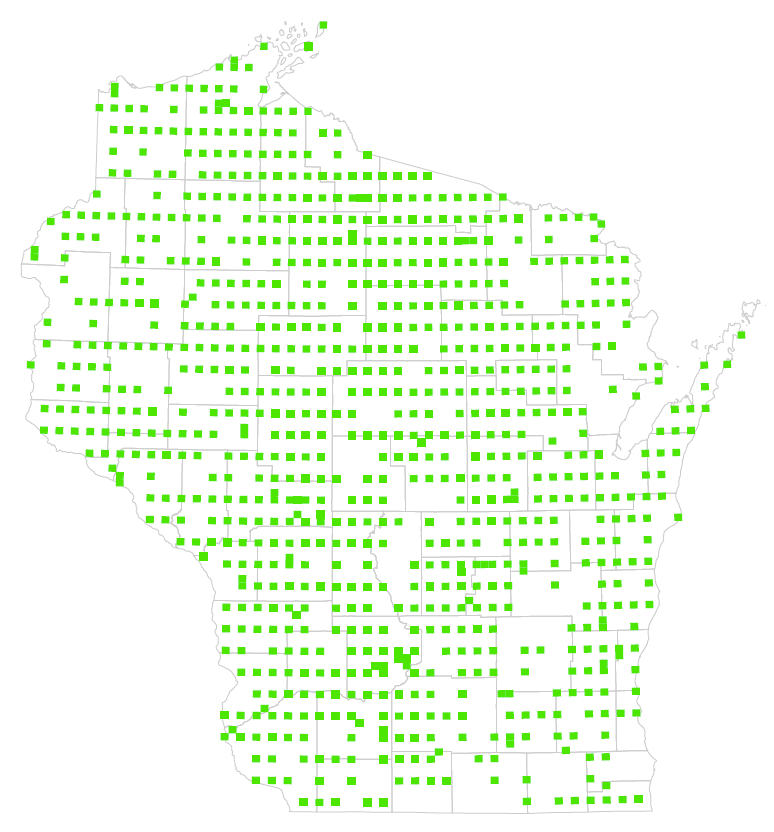


**Appendix D.** Comparable block set that indicates blocks with high levels of survey coverage for both periods of the Wisconsin Breeding Bird Atlas. Comparable blocks were blocks that were deemed priority blocks during both atlas periods, and then blocks with a lopsided ratio of species between periods (outside 0.75 or 1.25) were discarded from this block set. This remaining set of blocks was used for the calculation of expanding or shrinking breeding ranges.

**
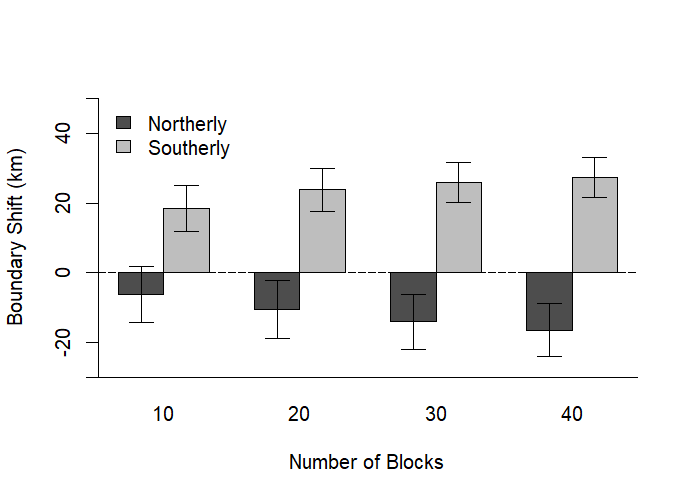
**

**Appendix E.** The resulting boundary shift (*y*-intercept) for different numbers of blocks comprising the boundary. Both the northerly (dark gray) and the southerly groups (light gray) had statistically similar boundary shifts between a boundary consisting of 10 v. 20 v. 30 vs 40 blocks. However, prior studies have used 10 latitudes to determine the boundary (Thomas & Lennon 1999; Brommer et al. 2012; Zuckerberg et al. 2009), as it best aligns with the concept of a range boundary. Additionally, multiple bird species did not have at least 20 detections to meet higher boundary standards, so using 10 latitudes to define the boundary is most inclusive of the species in question while also being directly comparable with prior studies.

**Appendix F.** Common name and scientific name of northerly species evaluated for range shifts between the first (1995–2000) and second (2015–2019) Wisconsin breeding bird atlases. **Boreal birds** are indicated with bold font.

| Species Common Name | Scientific Name |
| --- | --- |
| Trumpeter Swan | *Cygnus buccinator* |
| American Black Duck | *Anas rubripes* |
| Common Merganser | *Mergus merganser* |
| Ruffed Grouse | *Bonasa umbellus* |
| Common Loon | *Gavia immer* |
| Osprey | *Pandion haliaetus* |
| Sharp-shinned Hawk | *Accipiter striatus* |
| **American Goshawk** | *Astur atricapillus* |
| Bald Eagle | *Haliaeetus leucocephalus* |
| Broad-winged Hawk | *Buteo platypterus* |
| Yellow-bellied Sapsucker | *Sphyrapicus varius* |
| **Black-backed Woodpecker** | *Picoides arcticus* |
| Pileated Woodpecker | *Dryocopus pileatus* |
| Merlin | *Falco columbarius* |
| **Olive-sided Flycatcher** | *Contopus cooperi* |
| **Yellow-bellied Flycatcher** | *Empidonax flaviventris* |
| **Alder Flycatcher** | *Empidonax alnorum* |
| Least Flycatcher | *Empidonax minimus* |
| Blue-headed Vireo | *Vireo solitarius* |
| **Canada Jay** | *Perisoreus canadensis* |
| Common Raven | *Corvus corax* |
| **Boreal Chickadee** | *Poecile hudsonicus* |
| **Ruby-crowned Kinglet** | *Corthylio calendula* |
| Golden-crowned Kinglet | *Regulus satrapa* |
| Brown Creeper | *Certhia americana* |
| **Winter Wren** | *Troglodytes hiemalis* |
| Veery | *Catharus fuscescens* |
| **Swainson's Thrush** | *Catharus ustulatus* |
| Hermit Thrush | *Catharus guttatus* |
| **Evening Grosbeak** | *Coccothraustes vespertinus* |
| Purple Finch | *Haemorhous purpureus* |
| Dark-eyed Junco | *Junco hyemalis* |
| **White-throated Sparrow** | *Zonotrichia albicollis* |
| LeConte's Sparrow | *Ammospiza leconteii* |
| **Lincoln's Sparrow** | *Melospiza lincolnii* |
| Ovenbird | *Seiurus aurocapilla* |
| **Northern Waterthrush** | *Parkesia noveboracensis* |
| Golden-winged Warbler | *Vermivora chrysoptera* |
| Black-and-white Warbler | *Mniotilta varia* |
| Nashville Warbler | *Leiothlypis ruficapilla* |
| **Connecticut Warbler** | *Oporornis agilis* |
| Mourning Warbler | *Geothlypis philadelphia* |
| **Cape May Warbler** | *Setophaga tigrina* |
| Northern Parula | *Setophaga americana* |
| **Magnolia Warbler** | *Setophaga magnolia* |
| **Blackburnian Warbler** | *Setophaga fusca* |
| Chestnut-sided Warbler | *Setophaga pensylvanica* |
| Black-throated Blue Warbler | *Setophaga caerulescens* |
| **Palm Warbler** | *Setophaga palmarum* |
| Pine Warbler | *Setophaga pinus* |
| Yellow-rumped Warbler | *Setophaga coronata* |
| Black-throated Green Warbler | *Setophaga virens* |
| **Canada Warbler** | *Cardellina canadensis* |

**Appendix G.** Common name and scientific name of southerly species evaluated for range shifts between the first (1995–2000) and second (2015–2019) Wisconsin breeding bird atlases.

| Species Common Name | Scientific Name |
| --- | --- |
| Wild Turkey | *Meleagris gallopavo* |
| Ring-necked Pheasant | *Phasianus colchicus* |
| Yellow-billed Cuckoo | *Coccyzus americanus* |
| Sandhill Crane | *Antigone canadensis* |
| Red-shouldered Hawk | *Buteo lineatus* |
| Eastern Screech-Owl | *Megascops asio* |
| Red-headed Woodpecker | *Melanerpes erythrocephalus* |
| Red-bellied Woodpecker | *Melanerpes carolinus* |
| Acadian Flycatcher | *Empidonax virescens* |
| Willow Flycatcher | *Empidonax traillii* |
| Bell's Vireo | *Vireo bellii* |
| Yellow-throated Vireo | *Vireo flavifrons* |
| Tufted Titmouse | *Baeolophus bicolor* |
| Horned Lark | *Eremophila alpestris* |
| Purple Martin | *Progne subis* |
| Blue-gray Gnatcatcher | *Polioptila caerulea* |
| Carolina Wren | *Thryothorus ludovicianus* |
| Wood Thrush | *Hylocichla mustelina* |
| House Finch | *Haemorhous mexicanus* |
| Grasshopper Sparrow | *Ammodramus savannarum* |
| Field Sparrow | *Spizella pusilla* |
| Lark Sparrow | *Chondestes grammacus* |
| Henslow's Sparrow | *Centronyx henslowii* |
| Orchard Oriole | *Icterus spurius* |
| Louisiana Waterthrush | *Parkesia motacilla* |
| Blue-winged Warbler | *Vermivora cyanoptera* |
| Prothonotary Warbler | *Protonotaria citrea* |
| Hooded Warbler | *Setophaga citrina* |
| Cerulean Warbler | *Setophaga cerulea* |
| Northern Cardinal | *Cardinalis cardinalis* |
| Dickcissel | *Spiza americana* |

**Appendix H.** Percentage of species showing significant changes on the three metrics. As a group, most boreal species are moving north with contracting breeding ranges within the state. Northerly species show mixed patterns as a group. Southerly species as a group also consistently show northward movement, and breeding range expansion.

|  |  | **Range Size** | **Boundary Shift** | | **Mean Latitude** | |
| --- | --- | --- | --- | --- | --- | --- |
| **Boreal Species** | | 10.0% expanding | 50.0% shifting north | | 35.0 % moving north |  |
|  |  | 45.0% contracting | 25.0% shifting south | | 0 .0% moving south |  |
|  |  |  |  |  |  |  |
| **Northerly Species** | | 18.9% expanding | 30.2% shifting north | | 39.6% moving north |  |
|  |  | 26.4% contracting | 32.1% shifting south | | 9.4% moving south |  |
|  |  |  |  |  |  |  |
| **Southerly Species** | | 48.4% expanding | 58.1% shifting north | | 32.3% moving north |  |
|  |  | 3.2% contracting | 3.2% shifting south | | 9.7% moving south |  |

**Appendix I.** 95% confidence intervals of the boundary shift and mean latitude shift of Northerly species.

| Species | Boundary Shift (95% CI) | Mean Latitude Shift (95% CI) |
| --- | --- | --- |
| Trumpeter Swan | (-162.02,-111.10) | (-32.41,13.89) |
| American Black Duck | (0.00,166.66) | (-18.51,37.03) |
| Common Merganser | (-27.77,13.88) | (9.26,37.03) |
| Ruffed Grouse | (13.84,32.40) | (27.77,41.67) |
| Common Loon | (-9.25,9.25) | (-9.25,4.62) |
| Osprey | (-124.99,-83.33) | (-50.93,-27.78) |
| Sharp-shinned Hawk | (4.64,97.21) | (0.00,37.03) |
| Northern Goshawk | (55.56,143.50) | (13.89,55.55) |
| Bald Eagle | (-41.67,-18.51) | (-64.80,-46.29) |
| Broad-winged Hawk | (-32.41,-18.52) | (-9.26,9.26) |
| Yellow-bellied Sapsucker | (-27.78,-0.01) | (-18.51,0.00) |
| Black-backed Woodpecker | (27.77,64.81) | (0.00,32.41) |
| Pileated Woodpecker | (-18.52,0.00) | (-4.64,13.88) |
| Merlin | (-268.49,-199.06) | (-64.80,-27.77) |
| Olive-sided Flycatcher | (-9.25,41.66) | (-9.26,13.89) |
| Yellow-bellied Flycatcher | (-60.18,0.00) | (-9.25,9.26) |
| Alder Flycatcher | (-18.52,4.62) | (0.00,18.51) |
| Least Flycatcher | (4.63,9.26) | (13.88,27.78) |
| Blue-headed Vireo | (-74.07,18.51) | (-4.63,13.88) |
| Canada Jay | (18.51,37.03) | (0.00,13.89) |
| Common Raven | (-55.56,-41.66) | (-18.52,-4.63) |
| Boreal Chickadee | (0.01,27.78) | (-13.88,23.14) |
| Ruby-crowned Kinglet | (-32.41,-4.63) | (-4.63,18.51) |
| Golden-crowned Kinglet | (-97.21,32.40) | (-4.63,9.27) |
| Brown Creeper | (-13.89,4.63) | (2.54,27.77) |
| Winter Wren | (-23.14,4.64) | (9.26,23.15) |
| Veery | (-27.78,-4.63) | (4.63,23.14) |
| Swainson's Thrush | (-9.25,87.95) | (23.15,69.44) |
| Hermit Thrush | (-32.41,-4.63) | (-4.63,9.25) |
| Evening Grosbeak | (41.66,55.55) | (-4.63,18.52) |
| Purple Finch | (-23.15,50.92) | (-0.01,13.88) |
| Dark-eyed Junco | (0.00,60.17) | (-4.62,27.77) |
| White-throated Sparrow | (-32.41,50.92) | (4.63,18.51) |
| LeConte's Sparrow | (-13.89,46.30) | (32.40,83.33) |
| Lincoln's Sparrow | (-87.96,-18.52) | (0.00,13.88) |
| Ovenbird | (-9.26,4.62) | (4.64,18.53) |
| Northern Waterthrush | (4.63,41.67) | (13.88,37.03) |
| Golden-winged Warbler | (37.03,64.81) | (9.26,27.77) |
| Black-and-white Warbler | (-13.89,37.04) | (0.01,13.89) |
| Nashville Warbler | (-18.52,32.40) | (4.63,18.52) |
| Connecticut Warbler | (0.01,185.17) | (0.00,41.66) |
| Mourning Warbler | (-13.88,4.63) | (13.89,32.40) |
| Cape May Warbler | (-41.66,-18.51) | (-13.88,13.88) |
| Northern Parula | (-226.83,-78.70) | (0.00,13.89) |
| Magnolia Warbler | (13.88,152.77) | (4.63,18.52) |
| Blackburnian Warbler | (9.26,92.58) | (-4.62,13.88) |
| Chestnut-sided Warbler | (-18.52,4.64) | (4.63,18.51) |
| Black-throated Blue Warbler | (-9.26,41.66) | (0.00,23.14) |
| Palm Warbler | (-101.84,-23.14) | (-13.88,4.64) |
| Pine Warbler | (-69.44,-9.26) | (-13.88,4.63) |
| Yellow-rumped Warbler | (-9.26,23.15) | (0.00,13.88) |
| Black-throated Green Warbler | (0.00,60.18) | (4.63,18.52) |
| Canada Warbler | (4.63,83.32) | (18.52,41.66) |

**Appendix J.** 95% confidence intervals of the boundary shift and mean latitude shift of Southerly species.

| Species | Boundary Shift (95% CI) | Mean Latitude Shift (95% CI) |
| --- | --- | --- |
| Wild Turkey | (101.84,115.73) | (60.18,78.70) |
| Ring-necked Pheasant | (4.63,87.96) | (37.03,60.18) |
| Yellow-billed Cuckoo | (-4.62,37.03) | (-9.26,13.89) |
| Sandhill Crane | (4.63,41.66) | (9.26,27.78) |
| Red-shouldered Hawk | (-23.14,18.51) | (-9.26,18.52) |
| Eastern Screech-Owl | (0.00,97.21) | (-9.26,13.89) |
| Red-headed Woodpecker | (13.89,64.80) | (-18.51,4.63) |
| Red-bellied Woodpecker | (83.32,101.84) | (23.15,41.66) |
| Acadian Flycatcher | (58.10,111.10) | (-13.89,9.26) |
| Willow Flycatcher | (-27.77,4.63) | (-13.89,4.63) |
| Bell's Vireo | (-4.63,18.52) | (-23.14,27.77) |
| Yellow-throated Vireo | (-9.25,9.26) | (-4.63,18.52) |
| Tufted Titmouse | (41.67,69.43) | (9.26,32.41) |
| Horned Lark | (-4.63,23.14) | (-9.25,9.27) |
| Purple Martin | (-78.69,-32.41) | (-23.15,4.63) |
| Blue-gray Gnatcatcher | (9.26,37.04) | (-13.88,4.63) |
| Carolina Wren | (87.95,148.13) | (-40.81,0.00) |
| Wood Thrush | (4.63,23.15) | (13.89,37.03) |
| House Finch | (0.00,13.89) | (-23.15,-9.25) |
| Grasshopper Sparrow | (-13.88,18.51) | (-4.63,23.15) |
| Field Sparrow | (4.62,46.29) | (-13.90,0.00) |
| Lark Sparrow | (111.10,134.24) | (-37.04,23.15) |
| Henslow's Sparrow | (37.03,152.76) | (-32.40,4.63) |
| Orchard Oriole | (41.66,69.44) | (9.26,37.04) |
| Louisiana Waterthrush | (-18.52,70.66) | (-46.30,4.63) |
| Blue-winged Warbler | (12.18,42.77) | (23.14,41.67) |
| Prothonotary Warbler | (9.26,74.06) | (-64.50,-9.26) |
| Hooded Warbler | (-14.52,27.77) | (-27.77,13.88) |
| Cerulean Warbler | (-32.40,18.52) | (-37.03,-0.01) |
| Northern Cardinal | (55.55,83.32) | (4.62,18.51) |
| Dickcissel | (69.44,134.24) | (27.77,50.93) |
